# Litter thickness limits the seed germination and seedling growth of *Rhododendron* plants

**DOI:** 10.7717/peerj.20756

**Published:** 2026-02-05

**Authors:** Zijing Niu, Yuefeng Wang, Xuechun Zhao

**Affiliations:** College of Animal Science, Guizhou University, Guiyang, China

**Keywords:** *Rhododendron* plants, Litter accumulation, Allelopathic, Regeneration capacity

## Abstract

Litter is considered a major constraint that affects the sexual reproduction and regeneration of populations of *Rhododendron* seedlings. In this study, we analyzed the effects of litter accumulation and its allelopathy on the regeneration potential of four *Rhododendron* species, and investigated the impacts of different litter amounts and coverage methods on *Rhododendron* seed germination through indoor pot experiments. The results showed that low amounts of litter promoted the rate of emergence of seedlings, while high amounts of litter inhibited this process. The rate of emergence of *Rhododendron* reached its maximum value when the amount of litter was between 200 and 400 g/m^2^ . The seeds of four species of *Rhododendron* began to germinate between days 7 and 8 of the experiment. The duration of germination for the four *Rhododendron* species ranged from 8 to 11 days. Extracts from different decomposition layers of litter had a “suppressive” effect or a “low promotion and high inhibition” effect on the germination rates of four species of *Rhododendron*. The litter extract inhibited the germination of *Rhododendron decorum* and *Rhododendron delavayi* at 2 mg/mL of extract, while it promoted the germination of *Rhododendron irroratum* and *Rhododendron agastum* seeds. Therefore, the presence of litter under *Rhododendron* shrubs in Northwest Guizhou Province, China, likely reduces the germination of the populations of *Rhododendron* and may limit their renewal through sexual reproduction. Thus, this study suggests that the presence of litter under *Rhododendron* shrubs in Northwest Guizhou Province may significantly limit their reproduction by seeds.

## Introduction

With over 1,200 species, *Rhododendron* L. (Ericaceae) is the largest genus of woody plants in the northern hemisphere (Chamberlain et al., 1996; Li et al., 2018). It is the dominant genus in subtropical mountainous evergreen broadleaved forests, mixed coniferous forests, coniferous forests, and dark coniferous forests. Dwarf *Rhododendron* shrubs can only grow above or close to the tree line (Feng, 1988). Northwest Guizhou Province, China, is a key distribution area for *Rhododendron* plants, with a total of six subgenera and more than 50 species. It accounts for approximately 50% of the *Rhododendron* plants in Guizhou Province. In particular, the Baili Rhododendron Nature Reserve, located at the junction of Qianxi, Dafang, and Jinsha counties, is the most representative (Zhang & Rhododendron, 1990). There are 43–45 species of *Rhododendron* plants (Chen et al., 2015), and the constructive species are primarily *Rhododendron delavayi*, *Rhododendron irroratum*, *Rhododendron agastum*, and *Rhododendron decorum*. These are the largest and continuously distributed natural communities of *Rhododendron* and typical representatives of medium- and low-altitude mountainous wild communities of *Rhododendron* in the world (Rong, Chen & Wang, 2009; Sosnovsky et al., 2017).

Owing to the complex terrain and frequent human activities in Northwest Guizhou Province, the pattern of plant distribution is uneven, and the populations of *Rhododendron* are mostly concentrated in suitable habitat patches that have a small niche width and are highly similar; resulting in simple population structures and weak anti-interference capabilities (Zhang et al., 2015). Although the propagation of *Rhododendron* plants in this region follows an obvious *R*-strategy (Maclean et al., 2017; Tonin, Gerdol & Wellstein, 2020), their reproduction is hindered by factors such as thick litter layers or insufficient understory light resources (Wang et al., 2020a). Consequently, the germination of *Rhododendron* seeds becomes challenging, and the reproduction of population is primarily driven by sprouting (Li & Chen, 2005). Owing to the lack of seedling regeneration through seed propagation (Niu & Yao, 2017), the number of seedlings in the *Rhododendron* population is very small, which is not conducive to the stability and development of the communities of *Rhododendron* plants (Wu et al., 2017; Wang et al., 2020a). The germination rates of *R. agastum* and *R. irroratum* are 50%, that of *R. delavayi* is 30%, while *R. decorum* only has a germination rate of 10% (Li et al., 2024).

Studies on the effects of litter on seed germination can be traced back to the 1960s (Tao, Xu & Li, 1987; Yang et al., 2010). Researchers have subsequently successively studied the effects of litter thickness (Liu et al., 2017), mechanical obstacles (Zimmerbeutel et al., 2024), and allelopathic effects (Jin et al., 2022) on the germination of seeds and growth of seedlings. There has been little research on how litter affects the germination and growth of *Rhododendron* seeds and the growth of seedlings. Additionally, field investigations revealed that the total litter biomass in *Rhododendron*-dominated communities in Northwest Guizhou ranges from 900 to 2,300 g/m^2^, with a thickness of 2.5–5.0 cm. The amount of undecomposed litter reaches 300–500 g/m² (observational data). We hypothesized that the thickness of litter may be a key factor that limits the sexual regeneration of *Rhododendron* populations. To test this hypothesis, we investigated the effects of litter on *Rhododendron* seed germination by controlling two critical variables: litter amount and coverage method. Specifically, this study examined how litter influences the germination of seeds from four dominant *Rhododendron* species in the Baili Rhododendron Nature Reserve, with the aim of providing a scientific basis for the natural renewal of *Rhododendron* populations in this reserve.

## Materials and Methods

### Study area

The plant communities used for the experiment were selected from the Baili Rhododendron Nature Reserve (27°08′30″–27°20′00″N and 105°45′30″–106°04′45″E), which is located Northwest Guizhou Province at the altitude of approximately 1,449–1,845 m. The subtropical plateau monsoon climate prevails in the region. The average annual temperature is 11.5 °C and the average annual precipitation is 1,000–1,100 mm. The frost-free period is approximately 250 days, and the average annual hours of sunshine are 967.2–1,287 h. The soil pH is 4.2–5.1. The zonal vegetation is primarily evergreen broad-leaved forest, while the vegetation in the study area is dominated by *Rhododendron* shrubs. The dominant constructive species are *R. delavayi*, *R. irroratum*, *R. agastum*, and *R. decorum*.

### Seed material and collection

In November 2023, four typical *Rhododendron* communities, including *R*. *delavayi*, *R*. *irroratum*, *R*. *agastum*, and *R*. *decorum*, were selected as fixed samples in the Baili Rhododendron Nature Reserve (Table 1).

**Table 1:** Brief description of sample plots.

Sample polt	Location	Altitude (m)	Canopy density (%)	Dominant species	Companion species
*R. delavayi*	E105°51′11.41″	1,699	90	*R. delavayi*	*R. agastum*
	N27°14′16.48″				
*R. irroratum*	E105°51′10.30″	1,681	95	*R. irroratum*	*R. delavayi*
	N27°14′14.35″				
*R. agastum*	E105°51′12.57″	1,722	90	*R. agastum*	*R. delavayi*
	N27°14′20.31″				
*R. decorum*	E105°51′13.77″	1,747	75	*R. decorum*	*Quercus aliena*
	N27°14′37.27″				

**Figure 1:**
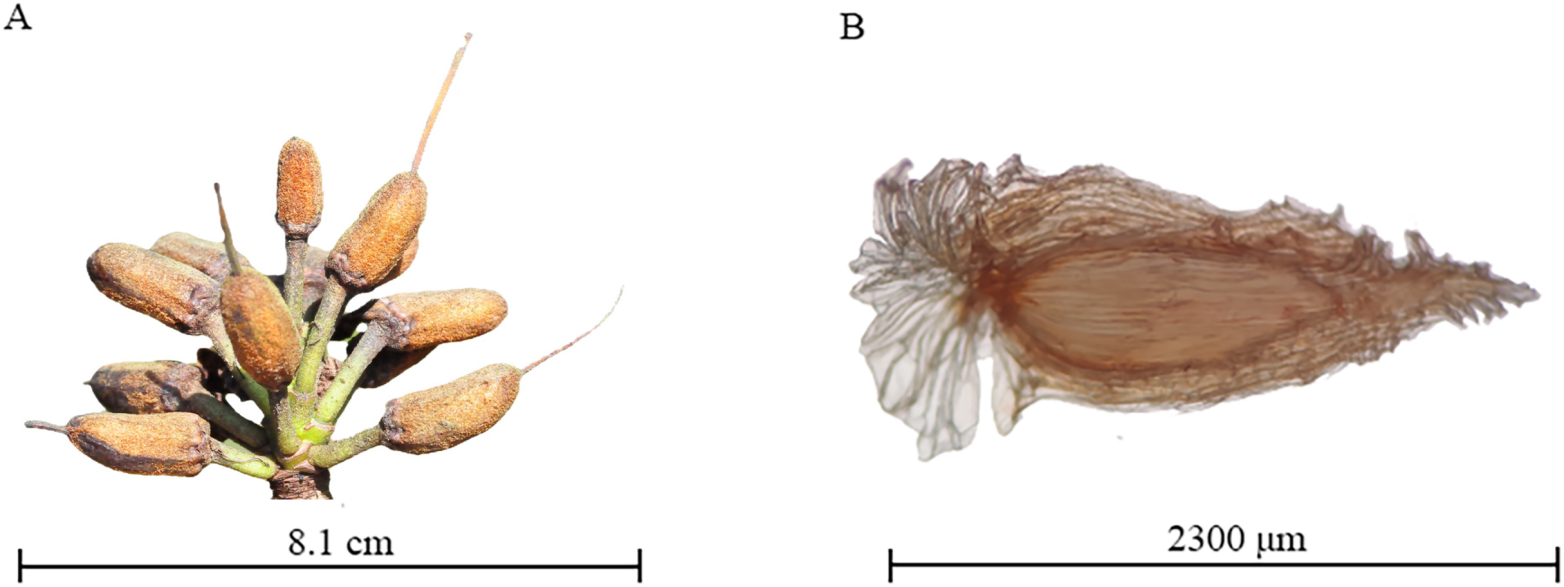
Demonstration of *Rhododendron* fruit and seed. (A) Fruit of *R. delavayi*; (B) seed of *R. irroratum*.

In November 2023, five plants each of *R*. *delavayi*, *R*. *irroratum*, *R*. *agastum*, and *R*. *decorum* were selected from the sample plots described above. Ten mature capsules (Fig. 1A) were taken from each plant, brought to the laboratory, cut open to retrieve the seeds (Fig. 1B), and then air-dried under natural conditions. The collected *Rhododendron* seeds were stored in a 4 °C refrigerator. Plump seeds with no pests or diseases were used for the seed germination and litter control experiment.

### Seed germination potted experiment

In March 2024, samples were collected from the litter layer (undigested fallen leaves of that year), soil layer (soil surface layer of 10 cm) in different *Rhododendron* communities. The samples were taken back to the laboratory, removed of impurities, and stored for future use.

**Figure 2:**
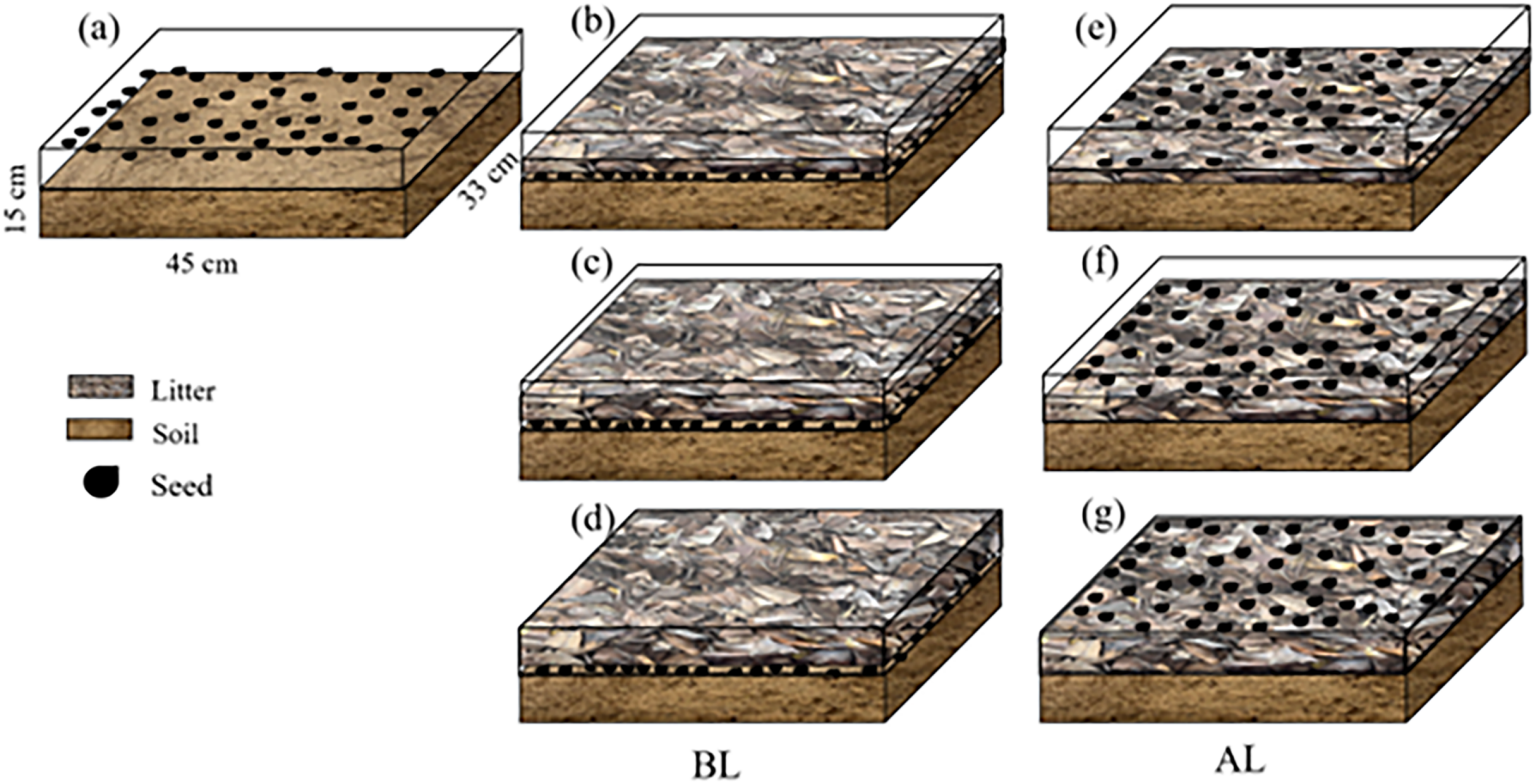
The litter treatments used in the study. (A) No litter (CK); (B), (C), and (D) seeds were placed below 200, 400, and 600 g/m^2^, of litter (BL), respectively; (E), (F) and (G) seeds were placed above 200, 400, and 600 g/m^2^ of litter (AL), respectively.

In March 2024, the fallen leaves that had been retrieved were cut to approximately 2 cm and stored for later use. A group of approximately 2/3 of nutrient soil (mixture of forest soil and the native humus layer) was placed in a rectangular plastic flowerpot that was 40.8 cm long and 25.5 cm wide. The bottom was 40.0 cm long and 25.0 cm wide, and the pots were 15.5 cm tall. Soil was filled into a flowerpot, 50 seeds were scattered evenly over the soil and covered with three litter amounts of 200, 400, and 600 g/m^2^ (BL) (Table 2). Another treatment was composed of soil in a flowerpot that was covered with three amounts of 200, 400, and 600 g/m^2^ litter (Zhang et al., 2017). A total of 50 seeds were then evenly sown above the litter (AL). Drip irrigation was used to water, keeping the soil and litter moist. There were four replicates, and soil without litter was used as the control (CK) (Fig. 2).

**Table 2:** Litter treatment design of different litter coverage methods and litter amount.

Litter coverage methods	Litter amount (g/m^2^)		
	200	400	600
No litter cover (CK)	—		
Litter covering seeds (BL)	BL1	BL2	BL3
The seeds are placed above the litter (AL)	AL1	AL2	AL3

### Effect of aqueous extract on seed germination

In July 2024, samples were collected from the litter layer (L layer, undigested fallen leaves of that year), humus layer (H layer), and soil layer (S layer, soil surface layer of 10 cm) in different *Rhododendron* communities. The samples were taken back to the laboratory, removed of impurities, and stored for future use. The collection of seeds, litter, humus, and soil was approved by the protected area’s management department and carried out in accordance with relevant local regulations.

In July 2024, at the Grassland Science Laboratory of Guizhou University (Guiyang, China) (106°39′28.03″E, 26°27′15.61″N), the samples from the litter layer, humus layer, and soil layer were dried, crushed, and laid separately in a cool place. They were then sieved through a 100-mesh sieve. Samples (litter, humus, and soil) were extracted with sterile distilled water at room temperature at a 1:10 ratio (sample: sterile distilled water) for 48 h. The supernatant was collected as the aqueous extract, with the concentrations of litter aqueous extract, humus aqueous extract, and soil aqueous extract all adjusted to 100 mg/mL. The aqueous extract was then serially diluted with sterile distilled water to yield final concentrations of 0, 2, 5, 10, and 20 mg/mL. Four levels of the aqueous litter extract were stored at 4 °C for further experiments.

The seed germination experiment was conducted using the filter paper culture dish method. The seeds were disinfected with 2% NaOH for 10 min and washed 5–6 times with sterile distilled water. They were then placed in culture dishes with 2 layers of filter paper. There were 50 seeds per culture dish, and the dishes were cultured in an incubator at 24 °C, 70% humidity, and 12 h of light. A volume of 3 mL of different concentrations of aqueous litter extract were added to the culture dish that contained the seeds (Zhou et al., 2015). An equal amount of corresponding concentration treatment solution was added every 2 days to maintain the humidity of the filter paper in the culture dish. There were four replicates for each treatment. The number of germinating seeds was recorded every 24 h.

### Data processing and statistical analysis

The seed germination rate (seedling emergence rate) was calculated using the following equation:

(1) $${G_r} = n/N \times 100\rm\%$$

where *G*_*r*_ is the germination (emergence) rate; *n* denotes the number of germinated seeds (emerged seedling), and *N* denotes the number of test seeds.

The allelopathic sensitivity index was calculated using the following equations:

(2) $$RI = 1 - C/T\; \left( {TC} \right)$$

(3) $$RI = C/T - 1\; (T < C)$$

where *RI* is the allelopathic sensitivity index; *C* is the control value, and *T* is the treatment value. *RI* > 0, promotion; *RI* < 0, inhibition, and the magnitude of the absolute value is consistent with the intensity of effect.

SPSS 26.0 (IBM Corp., Armonk, NY, USA) was used to analyze the data. The differences in the rates of germination and seedling emergence of different species of *Rhododendron* were tested by a one-way analysis of variance (ANOVA) using the Duncan method with a significance level of α = 0.05. A two-way ANOVA was used to examine the litter coverage method, litter amount and their interaction effects on seedling emergence after seed germination. Origin 2021 (OriginLab, Northampton, MA, USA) was used to draw the plots. All the data are presented as the mean ± SE.

## Results

### Effects of the amount of litter on the seedling emergence of different species of *Rhododendron*

**Figure 3:**
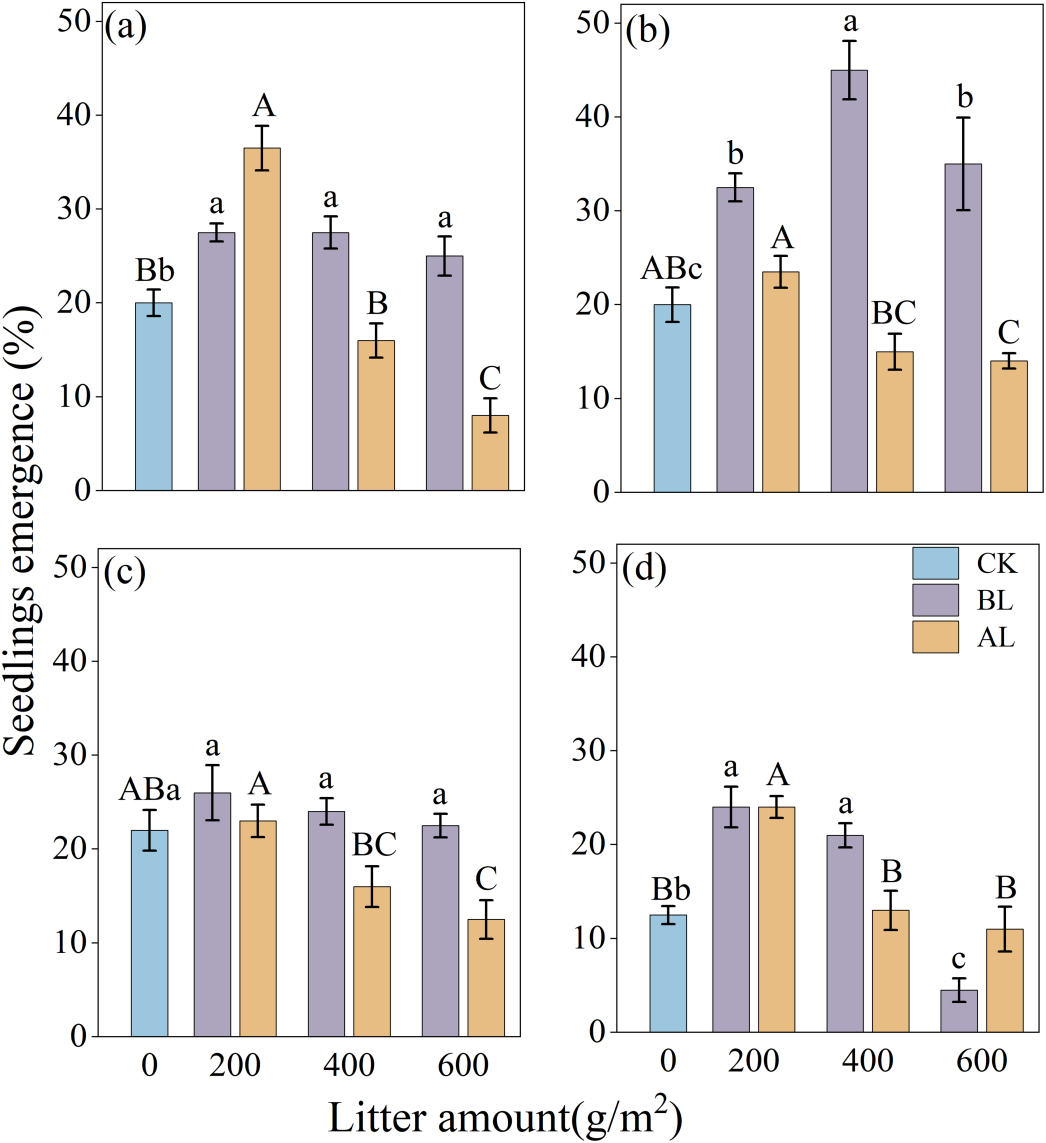
Effects of different amounts of litter and litter cover methods on the rate of emergence of *Rhododendron* seeds. (A), (B), (C) and (D), rates of emergence of *R*. *decorum*, *R*. *irroratum*, *R*. *agastum*, and *R*. *delavayi*, respectively. CK, direct sowing of the seeds; BL, seeds that were covered with litter; AL, seeds that were placed above the litter. Different lowercase letters, there were significant differences between BL and the rate of seedling emergence (*P* < 0.05). Different uppercase letters, the amount of litter placed above the litter (AL), and the rate of seedling emergence rate were significant (*P* < 0.05).

The effects of litter coverage and amount on the rate of emergence of *Rhododendron* seeds are shown in Fig. 3. Under two types of litter treatments, including covering the seeds with litter (BL) and placing them above the litter (AL), the rates of the seedling emergence of the four *Rhododendron* species (except *R. irroratum*) showed that, low litter promoted the emergence of seedlings, while high litter inhibited their emergence. A comparison of the two types of litter cover methods showed that except for the 200 g/m^2^ litter amount of *R*. *decorum*, the rate of emergence of *R*. *decorum* seedlings covered with litter (BL) was lower than that of those placed above the litter (AL). All the other treatments showed that the rate of emergence of *Rhododendron* seedlings covered with litter (BL) was higher than that of those placed above the litter (AL).

When the seeds were covered with litter (BL), the maximum rates of emergence of *R*. *decorum*, *R*. *agastum*, and *R*. *delavayi* were all achieved at 200 g/m^2^ of litter, with rates of emergence of 27.50%, 26.00%, and 24.00%, respectively. *R*. *irroratum* reached its maximum rate of emergence of 45.00% when the amount of litter reached 400 g/m^2^. When the seeds were placed above the litter (AL), the rate of emergence of the *Rhododendron* seedlings reached its maximum value at 200 g/m^2^, which was similar to the trend of BL treatment (Fig. 3).

The results of the two-factor ANOVA indicated that the method of covering the seeds with litter significantly affected the rates of seedling emergence of *R*. *decorum*, *R*. *irroratum*, and *R*. *agastum*. Additionally, the amount of litter had significant effects on the rates of seedling emergence of *R*. *decorum*, *R*. *agastum*, and *R*. *delavayi*. Furthermore, the interaction between the litter coverage method and the amount of litter significantly affected the rates of emergence of *R*. *decorum*, and *R*. *irroratum*, and *R*. *delavayi* (Table 3).

**Table 3:** F-statistics from the two factor ANOVA of the effects of litter coverage and litter amount on the emergence of *Rhododendron* seedlings.

Items	Species			
	*R. decorum*	*R. irroratum*	*R. agastum*	*R. delavayi*
Litter coverage methods (L)	19.790**	89.68**	17.844**	0.13
Litter amount (A)	38.812**	2.317	6.110*	46.122**
L × A	29.314**	8.295*	1.578	9.155*

**Notes:**

* *P* < 0.05.

** *P* < 0.01.

### Effect of the allelopathy of litter on the germination of *Rhododendron* seeds

**Figure 4:**
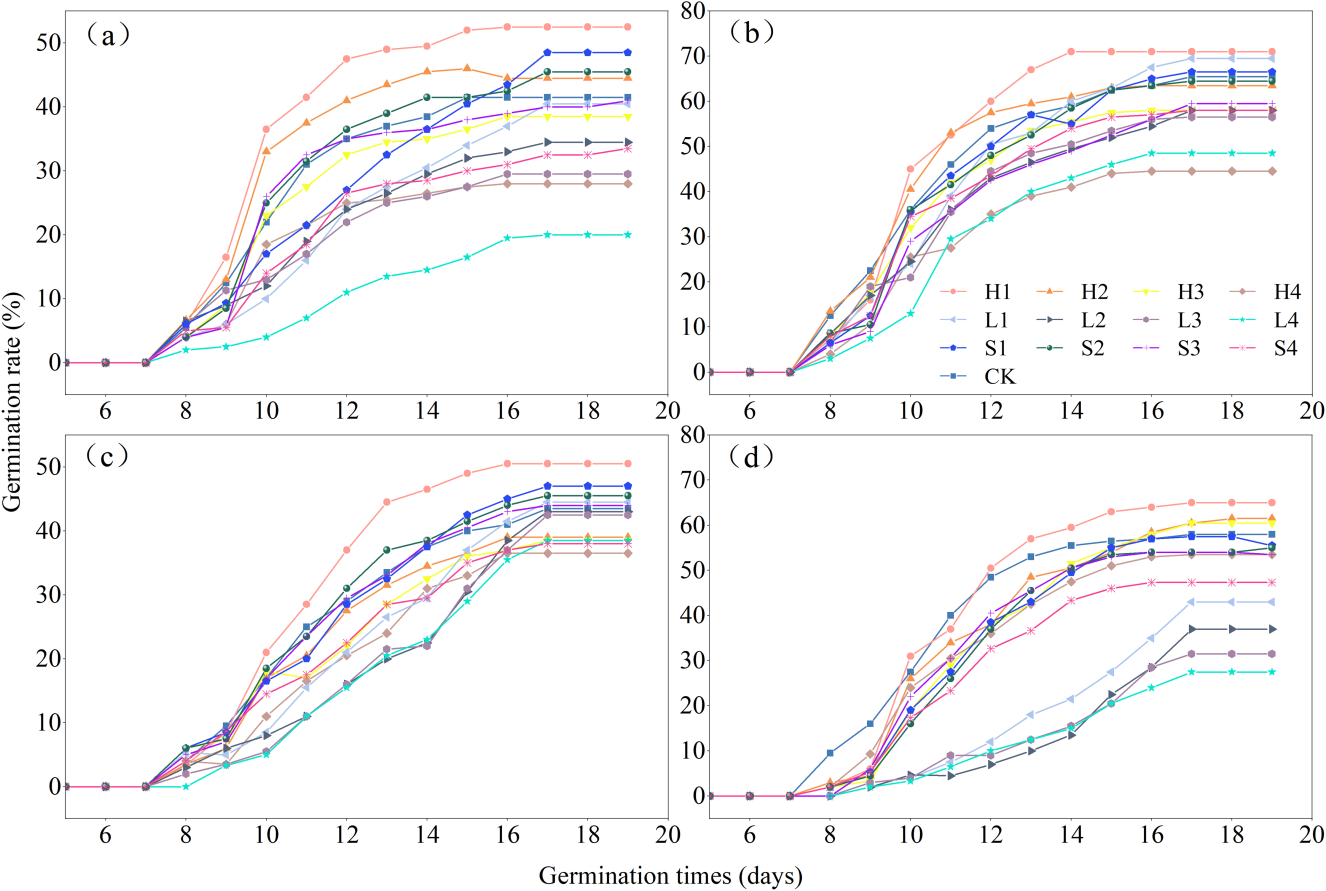
Effects of litter cover on the germination process of four *Rhododendron* seeds. (A)–(C), and (D) germination process of *R. decorum*, *R. irroratum*, *R. agastum*, and *R. delavayi*, respectively. L. H and S, litter, humus, and soil extract, respectively. 1, 2, 3, and 4, extraction concentrations of 2, 5, 10, and 20 mg/mL, respectively.

The effects of various types of extracts and concentrations on the process of germination of the four species of *Rhododendron* are shown in Fig. 4. Under different treatments, the germination rate of *Rhododendron* species initially increased and then gradually decreased over time. The seeds of all four species of *Rhododendron* began to germinate between days 7 and 8. Notably, *R*. *delavayi* germinated later than *R*. *decorum*, *R*. *irroratum*, and *R*. *agastum*, and it began to germinate on day 8. The duration of germination for the four species of *Rhododendron* ranged from 8 to 11 days, depending on the treatment.

**Figure 5:**
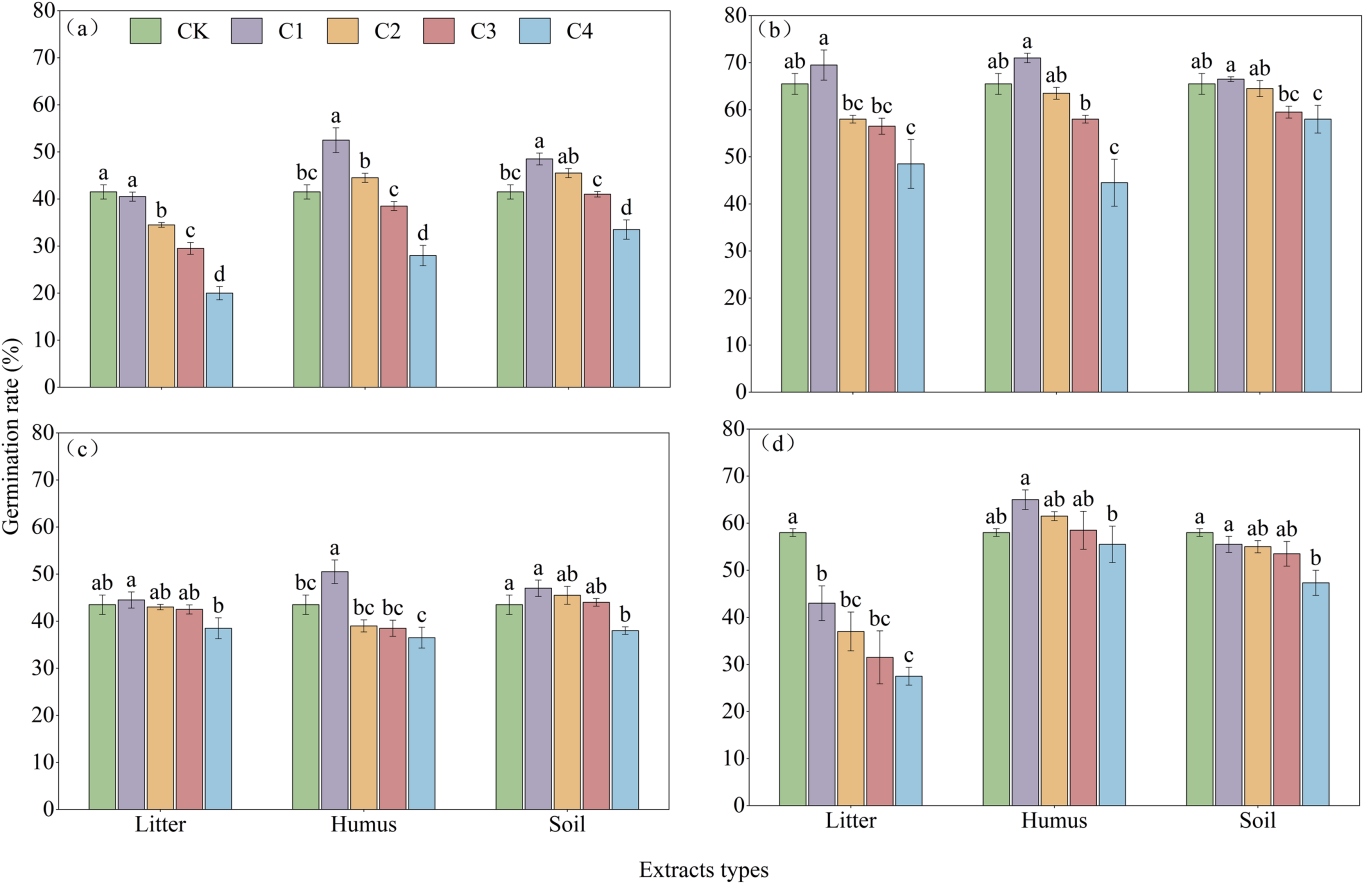
Effects of different types and concentrations of extracts on the germination rate of four seeds of *Rhododendron* species. CK, C1, C2, C3, and C4 represent sterile distilled water, with extraction concentrations of 2, 5, 10, and 20 mg/mL, respectively; (A)–(C), and (D), germination rates of seeds of *R*. *decorum*, *R*. *irroratum*, *R*. *agastum*, and *R*. *delavayi*, respectively. Different lowercase letters indicate significant differences (*P* < 0.05).

With the increase in the concentration of extraction solution, treating the seeds with litter extract decreased the germination rate of *R*. *decorum*, while the treatments of humus and soil of *R*. *decorum* seeds with humus and soil extracts initially increased the germination rate but then decreased it. Compared to the control (CK) with a germination rate of 41.50%, 2 mg/mL of litter extract had no significant effect and resulted in a germination rate of 40.50%. Treatments with 2 mg/mL of humus and soil extracts caused the highest germination rates of *R*. *decorum* that reached 52.50% and 48.50%, respectively (Fig. 5A). As the concentration of extract increased, the effects of litter, humus, and soil extracts on the germination rates of *R*. *irroratum* and *R*. *agastum* also tended to increase initially followed by a decrease. Compared to the CK (65.50%), the germination rates of *R*. *irroratum* seeds treated with 2 mg/mL of litter, humus, and soil extracts increased by 6.11%, 8.40%, and 1.53%, respectively (Fig. 5B). Similarly, compared to the CK (43.50%), the germination rates of *R*. *agastum* seeds treated with 2 mg/mL of litter, humus, and soil extracts increased by 2.30%, 16.09%, and 8.05%, respectively (Fig. 5C). As the concentration of extract increased, treatment with litter and soil extracts decreased the germination rate of *R*. *delavayi* seeds, while the humus extract caused an initial increase followed by a decrease. Compared to the CK (58.00%), treatment of the *R*. *delavayi* seeds with 2 mg/mL of litter and soil extracts decreased the germination rates by 25.86% and 4.31%, respectively, while the humus extract increased it by 12.07% (Fig. 5D).

The results of the regression equations indicated that except for the *R. delavayi* treated with humus extract, treatments with the different extracts significantly correlated with the germination rates of *R. decorum*, *R. irroratum*, *R. agastum*, and *R. delavayi* (Table 4).

**Table 4:** Trend of the effect of extracts concentration on the germination rate of different *Rhododendron* seeds.

Spices	type	Fitted equation	*R* ^2^
*R. decorum*	Litter	*y* = −1.143*x*^2^ − 0.829*x* + 41.714	0.923**
	Humus	*y* = −2.929*x*^2^ + 7.614*x* + 43.343	0.760**
	Soil	*y* = −2.179*x*^2^ + 6.634*x* + 42.343	0.754**
*R. irroratum*	Litter	*y* = −*x*^2^ − 0.700*x* + 67.000	0.530**
	Humus	*y* = −2.571*x*^2^ + 4.786*x* + 66.357	0.755**
	Soil	*y* = −0.571*x*^2^ + 0.086*x* + 66.057	0.407**
*R. agastum*	Litter	*y* = −0.643*x*^2^ + 1.371*x* + 43.514	0.251*
	Humus	*y* = −0.500*x*^2^ − 0.600*x* + 45.800	0.311*
	Soil	*y* = −1.357*x*^2^ + 4.029*x* + 43.686	0.492**
*R. delavayi*	Litter	*y* = −7.250*x* + 53.900	0.667**
	Humus	*y* = −1.393*x*^2^ + 4.421*x* + 59.214	0.157
	Soil	*y* = −0.564*x*^2^ − 0.033*x* + 57.361	0.420**

**Notes:**

* *P* < 0.05.

** *P* < 0.01.

The results of the two-factor ANOVA indicated that the type of extract had a significant effect on the germination rates of *R. decorum* and *R. delavayi*. The concentration of extract significantly affected the germination rates of all four species of *Rhododendron*, while the interaction between extract type and concentration had a significant effect on the germination rates of *R*. *irroratum* and *R*. *agastum* (Table 5).

**Table 5:** F-statistics from the two-way ANOVA for the types of litter extract and concentration effects on the germination rate of *Rhododendron* seeds.

Items	Species			
	*R. decorum*	*R. irroratum*	*R. agastum*	*R. delavayi*
Extract types (E)	68.585**	2.536	2.225	73.083**
Extract concentration (C)	102.303**	27.167**	16.599**	6.819**
T×C	1.878	2.524*	2.655*	0.493

**Notes:**

* *P* < 0.05.

** *P* < 0.01.

**Figure 6:**
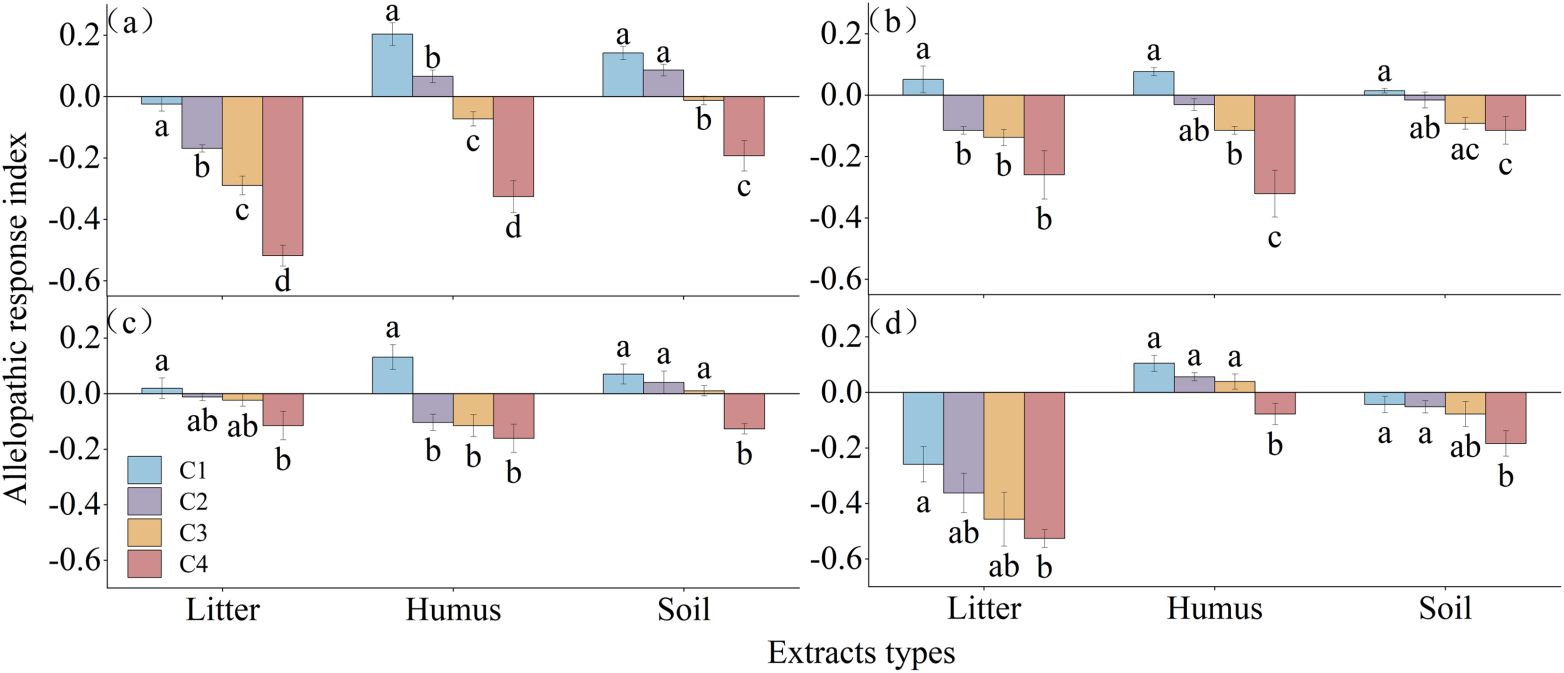
Allelopathic sensitivity index of *Rhododendron* seed germination under different extraction types and concentrations. C1, C2, C3, and C4, concentrations of 2, 5, 10, and 20 mg/mL of extract, respectively. (A)–(C), and (D), germination rates of seeds of *R*. *decorum*, *R*. *irroratum*, *R*. *agastum*, and *R*. *delavayi*, respectively.

The allelopathic sensitivity index of different extracts on the germination of *Rhododendron* seeds is shown in Fig. 6. The litter extract inhibited the germination of *R*. *decorum* and *R*. *delavayi*, while treatment with 2 mg/mL promoted the germination of *R*. *irroratum* and *R*. *agastum* seeds. The concentration of humus extract showed a pattern of promoting the germination of seeds at low concentrations and inhibiting germination at high concentrations for all four species of *Rhododendron*. Similarly, the soil extract promoted the germination of the seeds of *R*. *decorum*, *R*. *irroratum*, and *R*. *agastum* at low concentrations but inhibited the germination of *R*. *delavayi* seeds at high concentrations.

## Discussion

This study aimed to clarify the effect of the quantity of litter on the potential of four species of *Rhododendron* to regenerate. A high amount of litter was found to negatively affect the emergence of seedlings (Wang et al., 2022; Möhler et al., 2021). Litter played a crucial role during the stages of seed germination and seedling growth, and it influenced the seed germination and survival of seedlings through its effects on the microclimate, nutrient cycling, chemical allelopathy, and physical barriers created by the leaf litter (Molofsky & Augspurger, 1992; Kostel-Hughes, Young & Carreiro, 1998; Bosy & Reader, 1995).

Litter is the first physical environment that seeds encounter after their dispersal. The mechanical barrier effect of litter prevents seed-soil contact (Bewley & Black, 1994) and delays or inhibits the emergence of seedlings from the soil surface, which, in turn, reduces the rates of seed germination (Bosy & Reader, 1995; Olson & Wallander, 2002). In this study, the germination rates of the four *Rhododendron* seeds placed beneath the litter were higher than those of the seeds positioned on top of the litter. This is primarily because the litter possesses certain abilities to retain water (Zagyvai-Kiss et al., 2019), which can reduce the rates of evaporation of soil moisture, minimize temperature fluctuations, alter the dynamics of herbivory, and provide abundant nutritional resources; these create a favorable habitat for the seeds to germinate and seedlings to grow (López-Barrera & González-Espinosa, 2001). Regardless of whether the seeds were covered by litter or placed on top of it, the rates of emergence of the *Rhododendron* seedlings initially increased and then decreased with the amount of litter. This indicated that the excessive accumulation of litter negatively impacts the rates of seedling emergence, which is consistent with the findings of Liu et al. (2017). There are several possible explanations for this observation. First, the physical barrier created by a heavy cover of litter may deplete the energy reserves of seeds before the seedlings can emerge through the surface of litter, which leads to their death. Secondly, the horizontal orientation of the seedling cotyledons may hinder their ability to push upward through the dense litter. Third, the quantity and quality of light are reduced under a thick cover of litter could result in insufficient photosynthesis to meet the demands of seedlings for respiration (Rotundo & Aguiar, 2005; Donath & Eckstein, 2008).

The germination of seeds is the first stage of plant growth and development and a crucial step in the natural regeneration of plant communities (Bewley & Black, 1994; Du et al., 2007). The successful germination of seeds into seedlings determines the reproduction and survival of plant communities (Rajjou et al., 2012). Seed characteristics, temperature, moisture, light, and their interactions can all influence the germination of seeds (Glenn, Blazich & Warren, 1998; Hay, Klin & Probert, 2006; Mamo et al., 2006). *R. agastum* is a natural hybrid of *R. irroratum* and *R. delavayi* (Zha, Milne & Sun, 2009). the observed results are also associated with the genetic structure of these *Rhododendron* species (Fig. 3). The germination rates of different species of *Rhododendron* were low and varied significantly, ranging from 41.50% to 65.50%, with an average germination rate of 52.13%. This value was higher than the germination rates of 36 *Rhododendron* species in the subalpine region of the Tibetan Plateau, China (26.70% to 33.50%) (Wang et al., 2018a). The relatively low rates of germination of the *Rhododendron* seeds may be attributed to their small size (with a 1,000-seed weight of only 0.074 to 0.269 g), which results in less storage of nutrients (Zhao et al., 2015) and poorer abilities to germinate and establish seedlings (Bu et al., 2007; Chen et al., 2013; Kahmen & Poschlod, 2008).

Studies have shown that the germination of *Rhododendron* seeds is significantly influenced by the allelopathic effects of litter (Zhou et al., 2015). The allelochemicals produced by *Rhododendron* plants, including terpenes, naphthalene, phenolics, esters, alcohols, benzenes, organic acids, alkanes, and their derivatives, can inhibit the germination of their own seeds, as well as those of other plants in the community (Zhou et al., 2015; Nilsen et al., 1999). These findings are consistent with studies on the effects of litter from other species of *Rhododendron*, as well as those of *Celastrus orbiculatus*, *Cunninghamia lanceolata*, *Nyssa yunnanensis*, and *Toona ciliata* (Ellsworth, Harrington & Fownes, 2004; Wang et al., 2020b; Zhu et al., 2020; Zhang et al., 2016). In this study, different treatments of extracts from the layers of litter decomposition had inhibitory effects or dual effects of low promotion and high inhibition on different *Rhododendron* seeds. High concentrations of litter (5 mg/mL) inhibited the seed germination indicators, whereas low concentrations (2 mg/mL) had no significant effect or only slightly promoted germination. This outcome aligns with the “low promotion and high suppression” mass concentration effect observed in some autotoxic compounds on plant growth (Warrag, 1995; Wang, Wu & Jiang, 2018; Wang et al., 2018b; Huo et al., 2019; Zhang et al., 2020). This could be because low concentrations of autotoxic compounds activate the antioxidant defense mechanisms of the plants, which prompts them to implement strategies, such as increasing seed germination and growth rates, to adapt to adverse conditions (Craine & Dybzinski, 2013).

## Conclusions

This study revealed that the presence of a litter layer significantly hindered the potential of species of *Rhododendron* to regenerate. The litter layer acts as a barrier and source of allelopathy that prevents the seeds from reaching the soil, which leads to a reduction in their rate of germination. Thus, it can be concluded that the presence of a thick litter cover is one of the reasons why the population of *Rhododendron* in the Baili Rhododendron Nature Reserve has difficulty regenerating. Accordingly, to promote the germination capacity of *Rhododendron* seeds, it is advisable to conduct targeted and moderate clearance of litter in the habitat before the seeds reach physiological maturation.

## Supplemental Information

10.7717/peerj.20756/supp-1 Supplemental Information 1 Seed germination data under litter allelochemical treatment and Seed germination data under litter mechanical barrier treatment.

## Additional Information and Declarations

### Data Availability

The following information was supplied regarding data availability:

The raw data is available in the Supplemental File.
